# Bilhemia After Percutaneous Liver Tumor Core Biopsy With the Percutaneous Embolization of Bilio-venous Fistula With Coils and Onyx

**DOI:** 10.7759/cureus.61414

**Published:** 2024-05-31

**Authors:** Silva Breznik, Aleš Slanič, Arpad Ivanecz, Jernej Lučev

**Affiliations:** 1 Department of Radiology, University Medical Centre Maribor, Maribor, SVN; 2 Department of Abdominal and General Surgery, University Medical Centre Maribor, Maribor, SVN

**Keywords:** onyx embolization, embolization, liver biopsy, bilio-venous fistula, bilhemia

## Abstract

Bilhemia is a rare but potentially lethal condition representing abnormal communication and flow of bile into the bloodstream. We present a case of iatrogenic bilhemia after a percutaneous liver biopsy in a patient with cholangiocarcinoma. The bilio-venous fistula was visualized with percutaneous cholangiography and successfully embolized using coils and the liquid embolic agent Onyx. To our knowledge, this is the first report of using Onyx for the embolization of a bilio-venous fistula.

## Introduction

Communication between the bile ducts and the hepatic venous system can occur after liver trauma or different procedures on the bile ducts or liver. The most common form of this is hemobilia, which describes the flow of venous blood into the bile ducts since venous pressure is generally higher than biliary pressure. Normal pressure in the hepatic vein is 0-5 mmHg, in the common bile duct is 10-15 mmHg, in the hepatic artery is 100 mmHg, and in the portal system is 10 mmHg [1]. In cases where there is elevated pressure in the bile duct system over the hepatic or portal venous pressure, the flow of bile into the liver vein can occur and is referred to as bilhemia [2,3]. Many such communications close spontaneously due to slow flow through the bilio-venous fistula and may never be diagnosed [4]. Bilhemia becomes significant when a considerable amount of bile enters the bloodstream [4]. Usually, the obstruction of bile outflow with bile duct dilatation is the reason for bilhemia; however, cases of bilhemia without bile duct dilatation have been reported [3,5].

Bilhemia usually presents with jaundice and extremely elevated levels of bilirubin in the blood several days after the liver trauma. Bilhemia can also cause even more severe conditions such as hemolysis, biliary sepsis, and pulmonary embolism [1]. It is most dangerous when a large amount of bile suddenly enters the bloodstream and acts as an embolus in the lungs, potentially causing sudden death [4,6].

There are several possible causes, among which blunt abdominal trauma is thought to represent up to half of these cases [2,7]. Also, penetrating liver trauma, mostly of iatrogenic causes such as post cholangiopancreatography, post cholecystostomy, and different liver punctures, has been described as a causative factor for bilhemia [3,6,8-10].

If bilhemia does not resolve with conservative treatment, surgical, endoscopic, or percutaneous treatment procedures have been described in several case reports [3,6,11,12].

In the literature, there have been scarce reports of iatrogenic bilhemia caused by percutaneous core liver biopsy [1,3,13]. To our knowledge, the percutaneous embolization of bilio-venous fistula causing bilhemia has been described in only a few cases [8,14].

## Case presentation

**Figure 1:**
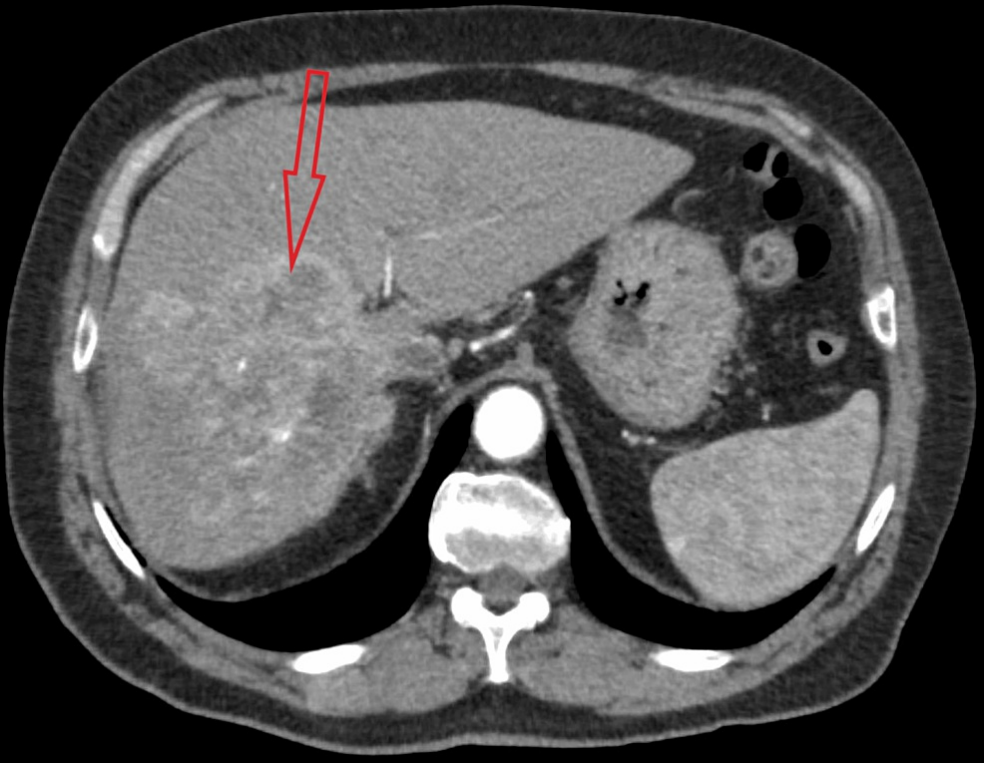
CT image of a tumor in the axial plane in the arterial phase (the red arrow points to the right liver lobe tumor) CT: computed tomography

**Figure 2:**
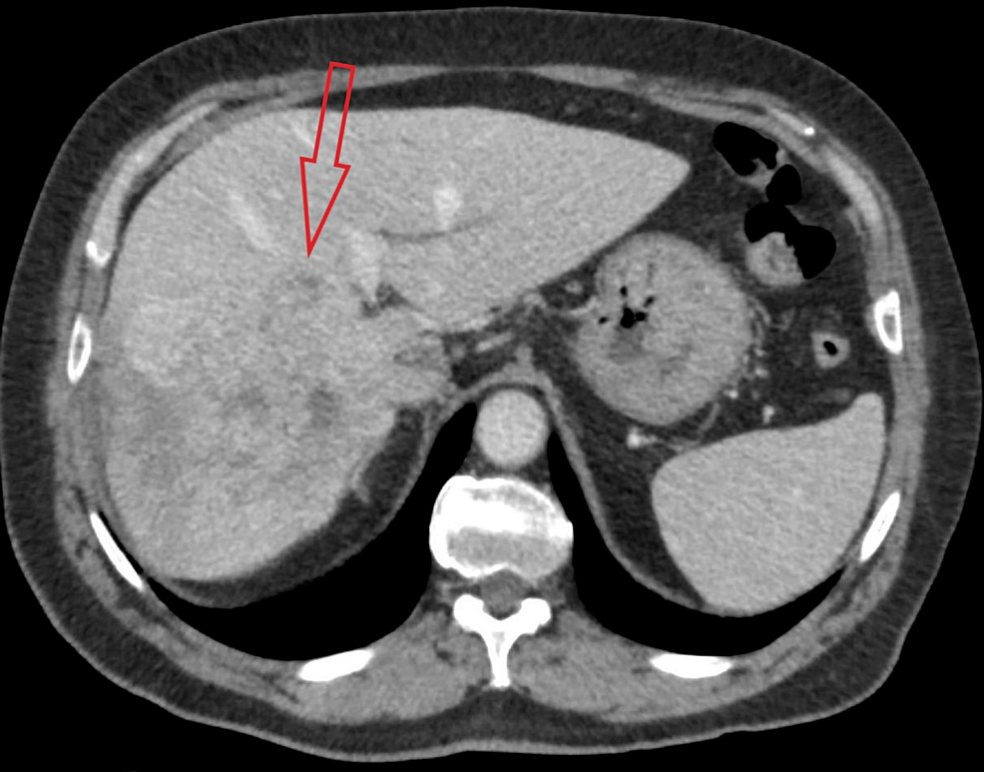
CT image of a tumor in the axial plane in the venous phase (the red arrow points to the right liver lobe tumor) CT: computed tomography

A 77-year-old male patient was admitted to our hospital for a planned percutaneous liver biopsy of a 12 × 11 cm mass (Figures 1, 2) infiltrating all segments of the right liver lobe. The mass had avid peripheral contrast enhancement in the arterial phase with some contrast washout and some necrotic areas (Figures 1, 2).

It was infiltrating the right and middle hepatic veins and abutting the inferior vena cava. The left liver lobe had been reactively hypertrophied, while all bile ducts were normal. A percutaneous tumor core needle biopsy in segment 4b bordering to segment 5 was performed with a subcostal approach using a coaxial technique with a 17 G trocar needle and 18 G core biopsy needle mounted on a Bard Magnum biopsy gun (New Providence, NJ). The procedure was uneventful, and the patient was discharged home.

Five days after the percutaneous core liver biopsy, the patient returned to the emergency department jaundiced with an extremely elevated conjugated bilirubin value of 1070 mmol/L (his prebiopsy bilirubin value was 14 mmol/L). Liver transaminases, cholestatic enzymes and pancreatic enzymes, and blood coagulation values were all within normal ranges. He also had pruritus and dark urine but denied any pain or encephalopathy. Computed tomography (CT) imaging did not show bile duct obstruction or any other new pathology in comparison to prebiopsy CT. A suspicion of biliary duct to systemic vein fistula was proposed. The biopsy needle most probably punctured into the middle hepatic vein causing bilio-venous fistula. Magnetic resonance imaging (MRI) of the liver with hepatospecific contrast medium (Primovist) and magnetic resonance cholangiopancreatography (MRCP) was performed but failed to show bilio-venous fistula on 30-minute postcontrast imaging due to delayed contrast excretion to bile ducts and the preliminary termination of the examination due to patient noncooperation.

**Figure 3:**
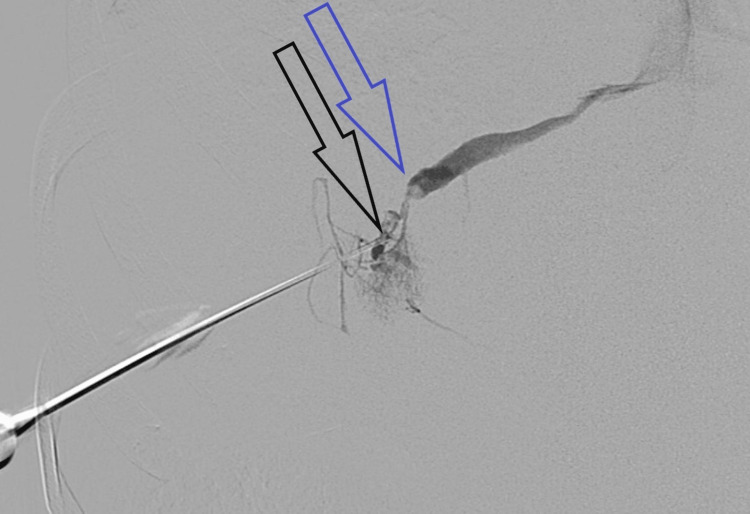
Bilio-venous fistula shown with percutaneous cholangiography (the blue arrow points to the middle hepatic vein, and the black arrow points to the puncture needle tip in the bile duct in segment 5; in between both is the bilio-venous fistula)

Because of the stagnant patient's clinical condition 19 days after percutaneous liver biopsy, percutaneous liver cholangiography was performed. The peripheral bile duct in segment 5 was punctured, and direct communication between the bile duct in segment 5 and the middle hepatic vein was confirmed (Figure 3).

A nonvascular 6 Fr micropuncture set (MAK-NV, Merit Medical, South Jordan, UT) was introduced over the 0.018 guidewire (Glidewire, Terumo, Somerset, NJ) 1.5 cm away from the bilio-venous fistula. Attempts to advance guidewire into central bile ducts for percutaneous biliary drainage had been unsuccessful. Based on the proximity and good visualization of the fistula, the decision for percutaneous closure of the fistula was made. A microcatheter (Progreat 2.7 Fr, Terumo) was advanced through the micropuncture sheath to the bilio-venous fistula but failed to cross the fistula.

**Figure 4:**
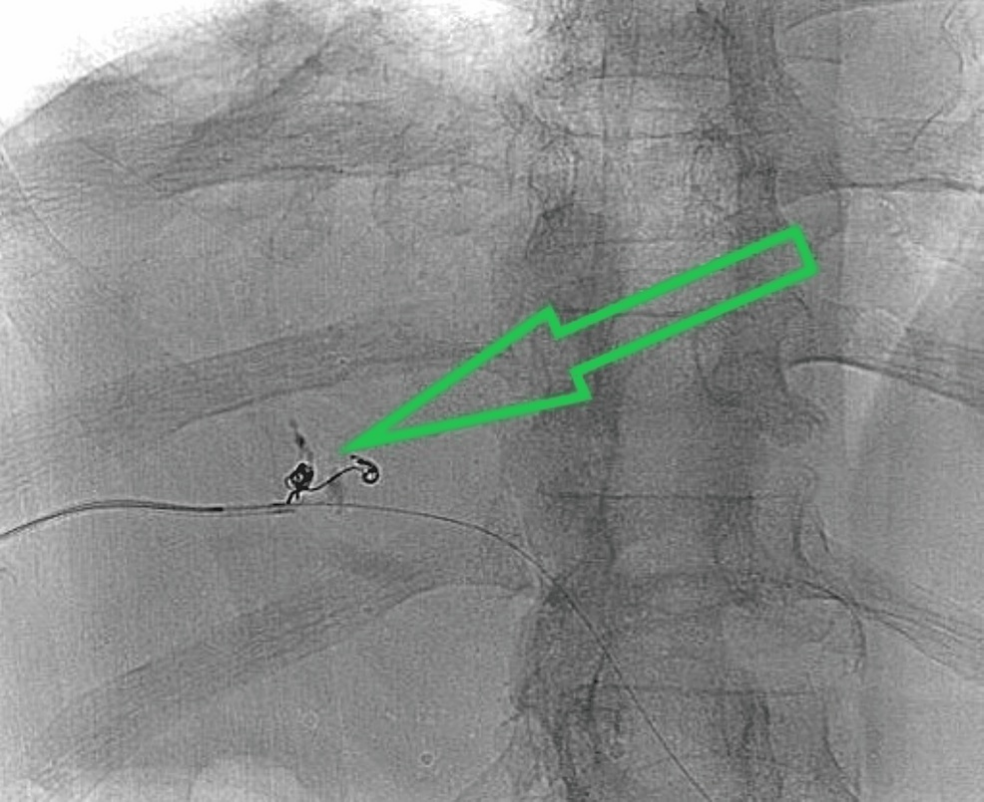
Embolization with coils failed to close the bilio-venous fistulas (green arrow)

**Figure 5:**
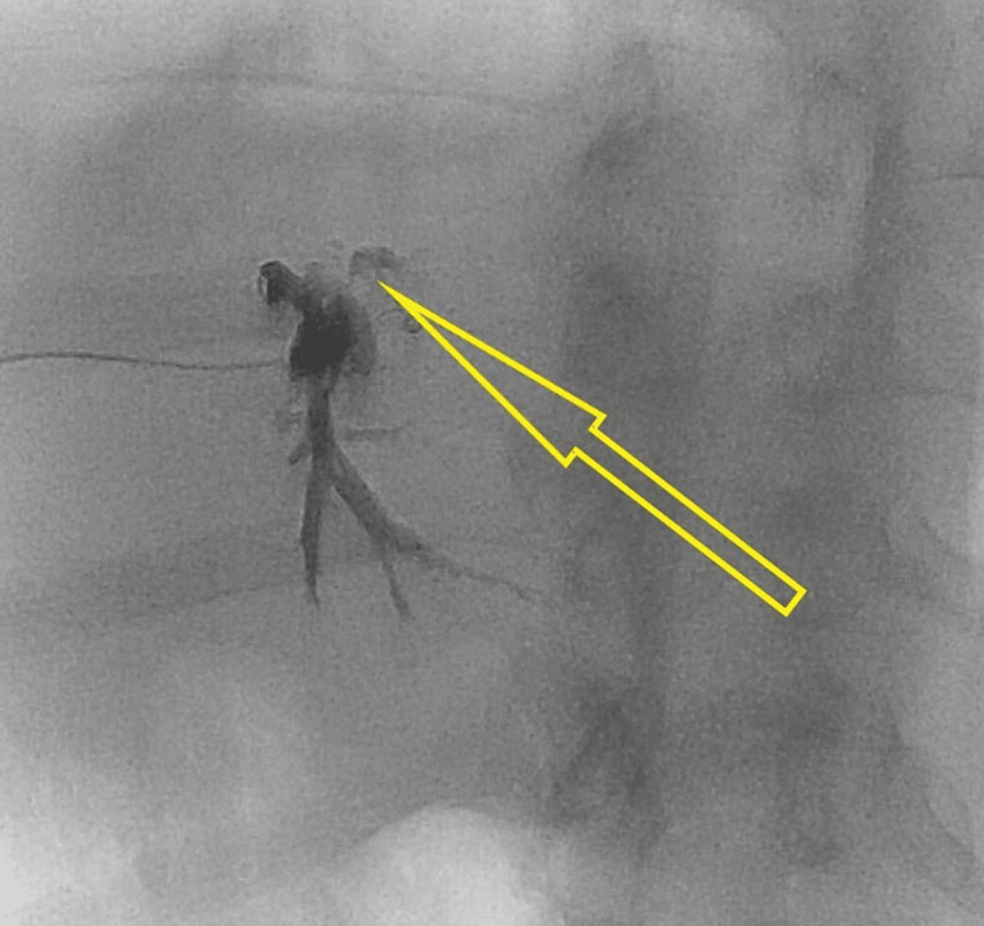
Embolization with Onyx closure of bilio-venous fistulas (yellow arrow)

Since embolization with two coils of diameters 4 mm and 20 cm long (Concerto, Medtronic, Minneapolis, MN) failed to close the communication (Figure 4), liquid embolization (Onyx, Medtronic) was performed subsequently with the positioning of the microcatheter in the vicinity of the bilio-venous fistula. The flow of Onyx away from the bilio-venous fistula was seen at first, causing some inadvertent embolization. After the repositioning of the microcatheter with withdrawal for a few millimeters, the flow of Onyx through the bilio-venous fistula into the middle hepatic vein was seen and resulted in the successful closure of the fistula (Figure 5). The 6 Fr catheter puncture tract was embolized with Gelfoam slurry.

In the following days, a rapid drop of bilirubin values was observed, but almost three months were needed to reach the prebiopsy level (15 mmol/L). By that time, the patient's clinical condition had also improved to the level that he tolerated extensive right liver lobe resection including segment 4. He was discharged from the hospital 19 days after liver resection with a percutaneous drain inserted into the biloma at the resection margin. Otherwise, his postoperative period has been uneventful.

The pathology examination revealed intrahepatic cholangiocarcinoma, which had probably evolved from dysplastic biliary adenofibroma.

## Discussion

Complications attributable to liver biopsy are rare, with a reported rate of up to 0.7% [14-16]. The most common complications of liver biopsy are bleeding, pneumothorax, bile peritonitis, pneumothorax, and hemobilia. Uncommon complications being reported are subcutaneous emphysema, pneumoperitoneum, subphrenic abscess, pancreatitis caused by hemobilia, and various bilio-venous fistulas [3]. The reported frequency of bilio-venous fistulas is 0.02%-0.1% [16].

The direction of flow in intrahepatic fistulas depends on pressure gradients. A supraphysiologic bilio-venous pressure gradient (more than 10 mmHg) is needed to cause clinically significant bilhemia. Fistulas with gradients of less than 10 mmHg should close spontaneously. Hepatic artery involvement decreases the likelihood of spontaneous closure of intrahepatic fistulas [17].

Iatrogenic causes described in the literature are bilhemia following transjugular or percutaneous liver biopsy [3,12,17] after transjugular intrahepatic portosystemic shunt (TIPS) procedure [5,6,9], percutaneous biliary drainage [14], and percutaneous liver ablation [18].

Since bilhemia represents a condition with high mortality, attempts to show abnormal communication with noninvasive imaging such as CT and MRI with MRCP have been used. In our case, all noninvasive diagnostic imaging failed, which is why the decision for the invasive procedure, percutaneous cholangiography, was made. The visualization of a bilio-venous fistula with endoscopic retrograde cholangiopancreatography (ERCP) has been described as a primary method for the demonstration of the direction of bile flow [4,6]. In our case, ERCP was not available at that time.

Different therapies to treat the leakage of bile into the veins have been described. When biliary dilatation is caused by an obstruction, the decompression of the biliary system with the drainage of bile external or internal to the bowel could allow the spontaneous closure of the existing bilio-venous fistula. Endoscopic drainage is associated with less morbidity and mortality compared to percutaneous and surgical biliary decompressions [3]. Internal drainage can be achieved by ERCP with/or without sphincterotomy, percutaneous internal-external drainage, and percutaneous or endoscopic stent/stentgraft insertion [6,11,12,19]. There have been scarce reports of surgical treatment of bilhemia after liver trauma [7].

In cases such as ours, without discernable biliary duct dilatation, the etiology of false negative might include acute obstruction, without bile ducts having ample time to dilate [20]. Conversely, in our case, the liver tumor was biopsied, and the inherent tumor structure could be the reason for the bilio-venous fistula, since no discernable bile duct dilatation has been present. By performing percutaneous cholangiography, we have demonstrated direct communication between the slightly dilated short segment of a bile duct and the middle hepatic vein, without being able to discern other parts of the bile duct system. Initially, after cholangiography, some unsuccessful attempts to navigate and pass to the central bile ducts to achieve percutaneous drainage have been made. Consequently, we have decided to perform percutaneous closure with the embolization of the bilio-venous fistula. While coil embolization was insufficient, since we were not able to cross the bilio-venous fistula with a microcatheter, liquid embolic Onyx was used, and the successful closure of the communication was achieved. To our knowledge, this is the first report of the treatment of a bilio-venous fistula with Onyx. The percutaneous embolization of a bilio-venous fistula can be performed from either the biliary or hepatic venous routes [3]. According to the literature, N-butyl cyanoacrylate glue has also been used as a liquid embolic agent [8], and a case of the closure of bilio-venous fistula with fibrin sealant using ERCP has been reported [12].

## Conclusions

Bilhemia is a rare and potentially lethal condition that occurs when the pressure in the bile ducts exceeds that of the venous system, resulting in extremely high levels of serum bilirubin.

We have confirmed the bilio-venous fistula with percutaneous cholangiography and successfully treated it with percutaneous embolization using coils and Onyx from the bile duct site of communication.
